# Individualized Nutritional and Microbiome-Oriented Management of Food Allergy-Associated Atopic Dermatitis in Children: A Prospective Real-World Observational Cohort Study

**DOI:** 10.3390/foods15162931

**Published:** 2026-08-21

**Authors:** Raluca-Gabriela Miulescu, Alina Mușetescu, Ruxandra-Cristina Marin, Călin Muntean, Radu Dumitru Moleriu, Alexandru-Neculai Pavel, Ioana Roșca, Laura Dinică, Alina Ilici, Oana Andreia Coman

**Affiliations:** 1 Faculty of Medicine, University of Medicine and Pharmacy “Carol Davila”, 020021 Bucharest, Romania; miulescuraluca@yahoo.com (R.-G.M.); ioana.rosca@umfcd.ro (I.R.); oana.coman@umfcd.ro (O.A.C.); 2 Dermatology Department, Saint Constantin Hospital, 500388 Brasov, Romania; 3 Department of Dermatology, “Dr. Victor Babeş” Clinical Hospital of Infectious and Tropical Diseases, 030303 Bucharest, Romania; alina.musetescu@gmail.com; 4 Department of Dermatology, “Titu Maiorescu” University, 031593 Bucharest, Romania; 5 Departament III Functional Science, Discipline of Medical Informatics and Biostatistics, “Victor Babes” University of Medicine and Pharmacy, 300041 Timisoara, Romania; radu.moleriu@umft.ro; 6 Pax Clinic, 020951 Bucharest, Romania; alexnpavel@yahoo.com; 7 Adeomed Clinic, 061344 Bucharest, Romania; medic@alergoped.ro (L.D.); drilici@yahoo.com (A.I.)

**Keywords:** atopic dermatitis, food allergy, gut–skin axis, gut dysbiosis, gut microbiome, elimination diet, personalized nutrition, probiotics, pediatric nutrition, real-world study

## Abstract

Atopic dermatitis (AD) and food allergy frequently coexist during early childhood and are increasingly linked through interactions within the gut–skin axis. We evaluated the clinical outcomes of an integrated nutritional and microbiome-oriented management strategy in children with food allergy-associated AD under real-world conditions. This prospective, two-center, real-world observational cohort study included 85 children (median age, 11 months) with AD diagnosed according to the Hanifin–Rajka criteria and confirmed IgE- and/or non-IgE-mediated food allergy. Participants received individualized nutritional and microbiome-oriented management comprising elimination diets, conventional topical therapy, microbiome-directed interventions, and nutritional supplementation tailored to their clinical, allergological, and microbiological profile. Disease severity was assessed using the Scoring Atopic Dermatitis (SCORAD) index at baseline and after approximately 1 and 3 months of follow-up. Cow’s milk, egg, and wheat were the predominant food allergens, and 69.4% of children were polyallergic. SCORAD decreased significantly during follow-up, with a mean relative reduction of 51.6% and 63.5% of participants achieving a SCORAD50 response. Food allergy burden independently predicted baseline disease severity, whereas documented gut dysbiosis was associated with polyallergy and greater absolute clinical improvement, although this association lost statistical significance after adjustment for baseline disease severity and should therefore not be interpreted as a favorable prognostic effect of dysbiosis. Baseline SCORAD remained the strongest independent predictor of clinical improvement. Integrated nutritional and microbiome-oriented management was associated with substantial clinical improvement and may represent a valuable adjunct to standard care in children with food allergy-associated AD. Because of the observational, uncontrolled design, these findings describe associations rather than causal treatment effects. These findings support further evaluation of personalized nutritional strategies in randomized controlled trials.

## 1. Introduction

Atopic dermatitis (AD) is the most common chronic inflammatory skin disease of childhood, affecting up to 20% of children in industrialized countries and frequently representing the earliest manifestation of the atopic march [1,2].

Its pathophysiology is multifactorial, involving epidermal barrier dysfunction, most notably filaggrin deficiency, type 2 immune dysregulation, and environmental and dietary factors that interact to sustain chronic cutaneous inflammation and pruritus [3].

Increasing recognition of the contribution of innate immune mechanisms to inflammatory skin diseases has further expanded our understanding of the complex immunopathogenesis underlying chronic dermatological disorders [4].

Loss-of-function variants in the FLG gene have been associated with increased disease severity and a higher risk of food allergy from early life, highlighting the close interplay between impaired barrier integrity and allergic sensitization [5].

Food allergy commonly accompanies AD during infancy and early childhood. Sensitization and clinical reactivity to cow’s milk, egg, and wheat are particularly frequent, and early-onset or more severe AD is associated with an increased risk of developing IgE-mediated food allergy and other manifestations of the atopic march [6,7].

Consequently, AD and food allergy should be regarded as closely interconnected disorders that share common mechanisms involving epithelial barrier dysfunction, immune dysregulation, dietary exposures, and alterations of the intestinal microbiota. Food allergy associated with AD encompasses IgE-mediated, non-IgE-mediated, and mixed phenotypes, each requiring distinct diagnostic and nutritional approaches [8,9].

Cow’s milk protein allergy remains the most common food allergy during early childhood and is a frequent trigger of eczema exacerbations [10].

Although elimination of confirmed food allergens remains a cornerstone of management, unnecessarily restrictive or poorly supervised elimination diets increase the risk of nutritional deficiencies and impaired growth, emphasizing the importance of individualized nutritional care [11,12].

Accordingly, nutritional management extends beyond simple allergen avoidance to include optimization of macro- and micronutrient intake, preservation of normal growth, support of epidermal barrier function, and modulation of immune responses through dietary interventions and the intestinal microbiota. Because allergic phenotype, nutritional requirements, gastrointestinal manifestations, and gut microbial composition vary substantially among children, personalized nutritional strategies are increasingly advocated to maximize clinical benefit while minimizing unnecessary dietary restrictions.

Over the past decade, the gut–skin axis has emerged as a central conceptual framework linking intestinal microbial homeostasis with cutaneous immune regulation [13]. Children with AD consistently exhibit gut microbial dysbiosis characterized by reduced abundance of short-chain-fatty-acid-producing bacteria, including Bifidobacterium and Lactobacillus, together with a relative expansion of proteolytic microorganisms. These alterations may precede or parallel disease activity and contribute to impaired immune tolerance [14,15]. Early-life factors influencing microbiome development, including cesarean delivery, prematurity, and the absence of breastfeeding, have also been associated with altered microbial colonization and an increased risk of allergic disease [16,17,18].

Biologically, microbial metabolites such as butyrate promote regulatory T-cell differentiation, strengthen epithelial barrier integrity, and contribute to immune tolerance, providing a plausible biological link between intestinal microbial composition and skin inflammation [19]. Recent evidence further indicates that disturbances in gut microbial composition and metabolic activity contribute to impaired epithelial barrier function, defective immune tolerance, and the development of both food allergy and AD, reinforcing the biological relevance of the gut–skin–food axis [20]. Short-chain fatty acids produced by bacterial fermentation of dietary fiber act on intestinal epithelial and immune cells—partly through G-protein-coupled receptors such as GPR43/FFAR2 and through inhibition of histone deacetylases—to promote regulatory T-cell differentiation, secretory IgA production, and tight-junction integrity; their deficiency favors increased epithelial permeability, systemic exposure to dietary antigens, and type 2-skewed immune responses [19,20]. In parallel, longitudinal birth-cohort data indicate that reduced gut microbial diversity during the first months of life precedes the clinical manifestation of AD, and that depletion of butyrate-producing taxa with enrichment of Enterobacteriaceae accompanies both eczema and food sensitization, supporting a temporal association and a plausible contributory role of early-life dysbiosis in subsequent allergic disease [14,15,16].

Although high-throughput sequencing technologies have substantially expanded our understanding of gut microbial ecology, standardized stool microbiology remains widely used in routine clinical practice to identify clinically relevant microbial imbalances and guide individualized nutritional and microbiome-oriented interventions. While culture-based methods cannot provide comprehensive taxonomic characterization, they offer pragmatic information that may support personalized clinical decision-making in real-world settings. These insights have motivated nutrition- and microbiome-oriented adjuvant strategies. Meta-analyses indicate that probiotics and synbiotics, especially multi-strain *Lactobacillus* and *Bifidobacterium* preparations, can reduce AD severity and SCORAD scores in children, while prebiotics such as inulin may favor a beneficial microbial milieu [21,22,23].

Similarly, vitamin D, through its immunomodulatory and barrier-supporting properties, and long-chain omega-3 polyunsaturated fatty acids (PUFA), through their anti-inflammatory and pro-resolving effects, have been investigated as nutritional adjuncts for pediatric AD, although the magnitude of their clinical benefit remains variable across studies [24,25].

All these findings have contributed to a growing shift toward personalized nutritional management. Rather than relying on isolated dietary interventions, individualized strategies increasingly integrate allergen elimination, microbiome-directed therapies, micronutrient optimization, and conventional dermatological treatment according to each child’s clinical phenotype, allergological profile, gastrointestinal manifestations, and microbiological characteristics. Because diet is one of the principal determinants of gut microbial composition, nutritional interventions provide an important opportunity to modulate immune responses through the gut–skin axis [26].

Despite this strong biological rationale, evidence from routine pediatric practice remains limited. Most published studies have evaluated individual nutritional interventions, including probiotics, prebiotics, vitamin D, or omega-3 supplementation, rather than comprehensive multimodal nutritional strategies implemented under real-world clinical conditions [1,27]. Furthermore, whether baseline gut dysbiosis influences the clinical response to individualized nutritional management remains insufficiently investigated.

Therefore, we conducted a real-world, two-center observational study including 85 children with food allergy-associated AD receiving individualized nutritional and microbiome-oriented management. The study aimed to characterize the demographic, perinatal, allergological, microbiological, and clinical profile of the cohort; identify factors associated with baseline disease severity; evaluate longitudinal changes in disease severity over approximately three months of follow-up; and determine whether gut dysbiosis and food allergy burden were associated with baseline disease severity and subsequent clinical response. By providing real-world evidence from routine pediatric practice, this study contributes to the growing field of personalized nutritional management within the gut–skin–food axis and may help inform the design of future interventional studies in children with food allergy-associated AD.

## 2. Materials and Methods

### 2.1. Study Design and Participants

This prospective, two-center, real-world observational cohort study included pediatric patients with atopic dermatitis (AD) and confirmed food allergy who received individualized nutritional and microbiome-oriented management during routine clinical practice at the Dermatology Department of Saint Constantin Hospital, Brașov, and the Adeomed Pediatric Clinic, Bucharest, Romania, between September 2025 and February 2026. Participants were followed longitudinally for approximately three months, with repeated assessments of disease severity at baseline, approximately 1 month, and 3 months. Children younger than 18 years with AD diagnosed by a dermatologist according to the Hanifin and Rajka criteria [28] and a confirmed IgE-mediated and/or non-IgE-mediated (delayed) food allergy were eligible.

Food allergy was established based on a compatible clinical history together with supportive investigations, including skin prick testing, atopy patch testing, and/or serum allergen-specific IgE measurement, with symptom improvement following elimination of the suspected food allergen; oral food challenge was not routinely performed in this real-world setting [9,29]. Consequently, the diagnosis of food allergy should be regarded as clinically probable rather than challenge-proven, and a degree of diagnostic misclassification cannot be excluded.

Exclusion criteria included other inflammatory or autoimmune skin diseases, active systemic infection, absence of confirmed food allergy, incomplete clinical data, and loss to follow-up before the first post-baseline assessment. A total of 85 children (aged 3–48 months) met the eligibility criteria and were included.

All clinical, allergological, and stool microbiological assessments were performed as part of routine clinical care. Prospectively collected clinical data were subsequently analyzed to evaluate longitudinal outcomes associated with individualized nutritional and microbiome-oriented management in a real-world setting.

### 2.2. Baseline Clinical, Allergological, and Gut Microbiological Assessment

At baseline, disease severity was assessed using the Scoring Atopic Dermatitis (SCORAD) index, a validated clinician-administered instrument that evaluates the extent and severity of AD, with higher scores indicating more severe disease [30,31].

SCORAD was calculated by a dermatologist using the dedicated scoring application. The SCORAD index combines three components: (A) the extent of the affected body surface area, estimated according to the rule of nines (0–100%); (B) the intensity of six clinical signs (erythema, edema/papulation, oozing/crusting, excoriation, lichenification, and dryness of uninvolved skin), each graded from 0 to 3; and (C) the subjective symptoms of pruritus and sleep loss, each rated on a 10 cm visual analogue scale. The total score is calculated as A/5 + 7B/2 + C, yielding values from 0 to 103, with disease classified as mild (<25), moderate (25–50), or severe (>50) [30,31]. Repeated assessments of the same child were performed by the same clinician throughout follow-up whenever feasible, thereby limiting potential inter-rater variability; however, formal inter-rater reliability testing was not performed. Food allergy was characterized using skin prick testing, atopy patch testing, and/or serum allergen-specific IgE measurement, with the diagnostic approach selected according to the clinical presentation and suspected immunological mechanism [29].

Skin prick testing was performed using standardized commercial food allergen extracts (Lofarma S.p.A., Milan, Italy), together with histamine dihydrochloride (10 mg/mL) as the positive control and glycerosaline as the negative control. A test was considered positive when the wheal diameter was ≥3 mm compared with the negative control. Extracts from the same manufacturer were used throughout the study to minimize methodological variability, as differences in allergenic potency among commercially available skin prick test products have been reported [32]. Antihistamines were discontinued for two weeks before testing whenever clinically appropriate to avoid false-negative results. Positive skin prick tests were interpreted in conjunction with the clinical history because sensitization alone does not establish clinical food allergy.

Atopy patch tests were performed according to the EAACI recommendations using standardized commercial food allergen preparations applied under occlusion to clinically uninvolved skin on the upper back for 48 h. Test reactions were evaluated immediately after patch removal and again after 72 h to support the diagnosis of delayed (non-IgE-mediated) food allergy [33,34].

Serum allergen-specific IgE concentrations were determined using commercially available immunoassays and interpreted together with the clinical history. Specific-IgE testing was particularly useful in children with extensive eczema or when skin testing was impractical; however, positive results were interpreted as evidence of sensitization rather than proof of clinical allergy. The final diagnosis of food allergy was established by an experienced pediatric allergist based on the clinical history and the results of the appropriate diagnostic investigations.

Stool microbiological testing was performed only in children with gastrointestinal manifestations suggestive of gut dysbiosis, including altered bowel habits, prolonged infantile colic, gastroesophageal reflux, or recurrent postprandial vomiting, which are frequently associated with food allergy and intestinal dysbiosis [35,36]. Because stool testing was performed only in symptomatic children rather than in the entire cohort, all dysbiosis-related findings should be regarded as exploratory and potentially affected by selection bias.

A single baseline stool sample was collected using dedicated transport containers and analyzed by a centralized reference laboratory in Germany using the same standardized clinical stool microbiology protocol for all samples. Samples were processed according to EUCAST standards using the Ganzimmun laboratory methodology. The laboratory performed a standardized, predominantly quantitative culture-based bacteriological and mycological analysis supplemented by physicochemical assessment, including quantitative culture of representative putrefactive (proteolytic) bacteria (*Escherichia coli*, *Proteus*, *Klebsiella*, *Enterobacter*, *Citrobacter*, and *Clostridium* spp.), protective (acidifying) bacteria (*Bacteroides*, *Bifidobacterium*, *Lactobacillus*, and *Enterococcus* spp.), fungal microorganisms (*Candida* spp., *Geotrichum*, and mould fungi), together with stool pH determination.

Based on the overall microbial profile, the laboratory generated a proprietary composite Flora Index integrating alterations in protective flora, putrefactive bacteria, fungal overgrowth, and stool pH, with higher values indicating greater gut dysbiosis. The Flora Index can therefore be interpreted as a single numerical measure of the degree to which a child’s cultivable gut flora deviates from the expected balanced pattern, with higher values indicating greater deviation. This clinically oriented assessment was used to document gut dysbiosis and guide individualized nutritional and microbiome-oriented management in routine practice. It does not represent a sequencing-based (16S rRNA or shotgun metagenomic) characterization of the intestinal microbiome, and the Flora Index should therefore be regarded as a laboratory-specific clinical indicator rather than an internationally standardized microbiome metric. Because the Flora Index is laboratory-specific, it was used only for descriptive and exploratory analyses in the subgroup of children who underwent stool testing.

Baseline demographic and clinical characteristics, including age, sex, gestational status (term or preterm), mode of delivery, conception by in vitro fertilization, and breastfeeding history, were also recorded.

### 2.3. Individualized Nutritional and Microbiome-Oriented Management

All children received an individualized nutritional and microbiome-oriented management strategy targeting the gut–skin–food axis. Management was centered on the elimination of individually identified food allergens, accompanied by nutritionally appropriate dietary substitution to maintain adequate nutrient intake. In formula-fed infants with cow’s milk protein allergy, substitution was based on extensively hydrolyzed or, when indicated, amino acid-based formulas in accordance with current guidelines [10,37], and caregivers received structured dietary counselling to ensure adequate energy, protein, calcium, and vitamin D intake. Elimination diets were restricted to foods with confirmed clinical relevance and were periodically re-evaluated to avoid unnecessary dietary restriction. Nutritional and microbiome-oriented support was tailored according to the clinical presentation and stool microbiological findings and included strain-specific probiotics or synbiotics (predominantly *Lactobacillus* and/or *Bifidobacterium* spp.; 95.3%), prebiotic inulin (23.5%), vitamin D_3_ (78.8%), omega-3 polyunsaturated fatty acids (28.2%), and correction of documented iron (10.6%) or zinc (3.5%) deficiencies. Antiparasitic therapy (3.5%) was prescribed when clinically indicated.

Standard dermatological management was provided according to disease severity and included emollients (91.8%), topical corticosteroids (25.9%), topical calcineurin inhibitors (29.4%), and oral antihistamines for pruritus control (47.1%). To account for potential confounding, the use of topical corticosteroids, topical calcineurin inhibitors, oral antihistamines, vitamin D_3_, omega-3 polyunsaturated fatty acids, and prebiotic inulin was recorded as binary covariates in the multivariable analyses. Probiotic supplementation was not modeled because it was prescribed to nearly all participants (95.3%).

### 2.4. Outcomes, Follow-Up and Data Management

Disease severity was assessed using the Scoring Atopic Dermatitis (SCORAD) index at baseline before initiation of individualized management, after approximately one month, and after approximately three months of follow-up. The primary outcome was the longitudinal change in SCORAD over the study period. Secondary outcomes included the absolute change from baseline to three months (ΔSCORAD), relative percentage improvement, achievement of a SCORAD50 response (defined a priori as a ≥50% reduction in SCORAD from baseline), indicating at least a halving of the overall disease-severity score, and changes in disease severity categories. Baseline disease severity was classified as mild (SCORAD < 25), moderate (25–50), or severe (>50). The number of implicated food allergens was recorded for each participant, and children were classified as monoallergic (one food allergen) or polyallergic (two or more food allergens).

The dataset underwent structured quality control before statistical analysis. Complete SCORAD assessments were available for all participants; therefore, no imputation was required for the primary outcome. Age was harmonized to months before analysis, and sensitivity analyses were performed to evaluate the influence of missing dysbiosis status, data coding decisions, and duplicated participant identifiers. Unless otherwise specified, analyses were conducted using complete-case data.

### 2.5. Statistical Analysis

Continuous variables are presented as mean ± standard deviation (SD) or median (interquartile range, IQR), as appropriate, and categorical variables as frequencies and percentages. Normality was assessed using the Shapiro–Wilk test. Associations between baseline characteristics and disease severity were evaluated using Spearman’s rank correlation, the Mann–Whitney U test, the Kruskal–Wallis test, and the χ^2^ or Fisher’s exact test, as appropriate. Exploratory univariate analyses were adjusted for multiple comparisons using the Benjamini–Hochberg false discovery rate (FDR) procedure.

Longitudinal changes in SCORAD were primarily analyzed using linear mixed-effects models with random intercepts for participants and fixed effects for assessment time, gut dysbiosis, and their interaction. Friedman and Wilcoxon signed-rank tests were performed as sensitivity analyses.

Multivariable analyses were performed to identify factors associated with baseline disease severity, SCORAD at three months after adjustment for baseline severity, absolute improvement in SCORAD (ΔSCORAD), and achievement of a SCORAD50 response. Models were adjusted for clinically relevant demographic, allergological, and treatment-related variables, including baseline disease severity (where appropriate), gut dysbiosis, age, sex, number of food allergens, and concomitant topical anti-inflammatory therapy. Standard and Firth’s penalized logistic regression were used for the SCORAD50 analysis because of the limited number of outcome events. Additional exploratory analyses evaluated factors associated with documented gut dysbiosis and the contribution of nutritional adjuvant therapies to clinical improvement.

The robustness of the principal findings was assessed using bootstrap resampling (5000 replicates) and sensitivity analyses. Statistical significance was defined as a two-sided *p*-value (or *q*-value for FDR-adjusted analyses) <0.05. All statistical analyses were performed using JASP version 0.18.3 (JASP Team, University of Amsterdam, Amsterdam, The Netherlands), with R version 4.3 serving as the computational backend.

### 2.6. Ethical Considerations

The study was conducted in accordance with the ethical principles of the Declaration of Helsinki. Ethical approval was obtained from the Institutional Review Board (Ethics Committee) of Saint Constantin Hospital, Brașov, Romania (approval no. 350 from 27 August 2025). In addition, institutional authorization for the use of anonymized clinical data from the participating ADEOMED Medical Clinic, Bucharest, Romania, was granted by the Medical Director (registration no. 30 from 27 August 2025).

Written informed consent was obtained from the parents or legal guardians of all participants before enrollment. Participation was voluntary, and caregivers were informed that refusal to participate or withdrawal from the study at any stage would not affect the medical care received. All clinical and microbiological data were anonymized before analysis to ensure participant confidentiality and data protection.

Safety outcomes were not predefined study endpoints, and adverse events were not assessed using a standardized reporting instrument. However, caregivers were routinely questioned about treatment-related adverse effects during follow-up visits, and no adverse events were reported during the study period. Formal safety evaluation should be addressed in future controlled studies.

## 3. Results

### 3.1. Characteristics of the Study Population and Food Allergy Profile

The study included 85 children with atopic dermatitis and confirmed food allergy, predominantly infants and toddlers (mean age 15.9 ± 12.4 months; median 11.0 months, IQR 6.0–24.0), with 96.5% aged ≤ 3 years. Boys accounted for 52.9% of the cohort. Cesarean delivery (74.1%), previous breastfeeding (57.6%), and documented gut dysbiosis (27.1%) were common, while baseline disease was predominantly of moderate severity (mean SCORAD 29.1 ± 10.4), with 56.5% of children classified as having moderate AD (Table 1).

Cow’s milk protein (92.9%), egg (57.6%), and wheat/gluten (37.6%) were the most frequently implicated food allergens, followed by tree nuts (14.1%) and potato (12.9%). Number of implicated food allergens (mean ± SD) was 2.5 ± 1.4, and polyallergy was more common than monoallergy (69.4% vs. 30.6%). Most children presented with a non-IgE-mediated (delayed) food allergy (60.0%), whereas 23.5% had an IgE-mediated pattern and 16.5% a mixed phenotype (Table 2).

These baseline characteristics provided the framework for subsequent analyses examining the relationships between food allergy burden, gut dysbiosis, and disease severity.

### 3.2. Determinants of Baseline Disease Severity

To identify factors associated with baseline disease severity, univariate and multivariable analyses were performed. Among the continuous variables evaluated, the number of implicated food allergens correlated positively with baseline SCORAD (Spearman’s ρ = 0.31, *p* = 0.004), remaining significant after Benjamini–Hochberg correction (q = 0.029). Neither age (ρ = 0.02, *p* = 0.84) nor the Flora Index within the dysbiosis subgroup (ρ = 0.10, *p* = 0.64) was associated with baseline disease severity. In unadjusted analyses, children with documented gut dysbiosis had higher baseline SCORAD scores than those without dysbiosis (median 32.0 vs. 26.2; Mann–Whitney U, *p* = 0.044), and disease severity categories differed according to dysbiosis status (χ^2^ = 6.39, *p* = 0.041). However, these associations were no longer significant after adjustment for multiple comparisons (q = 0.11). Mode of delivery, prematurity, and breastfeeding history were not associated with baseline disease severity (all *p* > 0.10).

In the multivariable linear regression model (R^2^ = 0.17), the number of implicated food allergens was the only independent factor associated with baseline SCORAD, with each additional implicated food allergen corresponding to a 2.19-point increase in SCORAD (95% CI 0.55–3.82, *p* = 0.009). None of the remaining covariates showed an independent association with baseline disease severity (Table 3).

### 3.3. Clinical Outcomes Following Nutritional and Microbiome-Oriented Management

**Figure 1:**
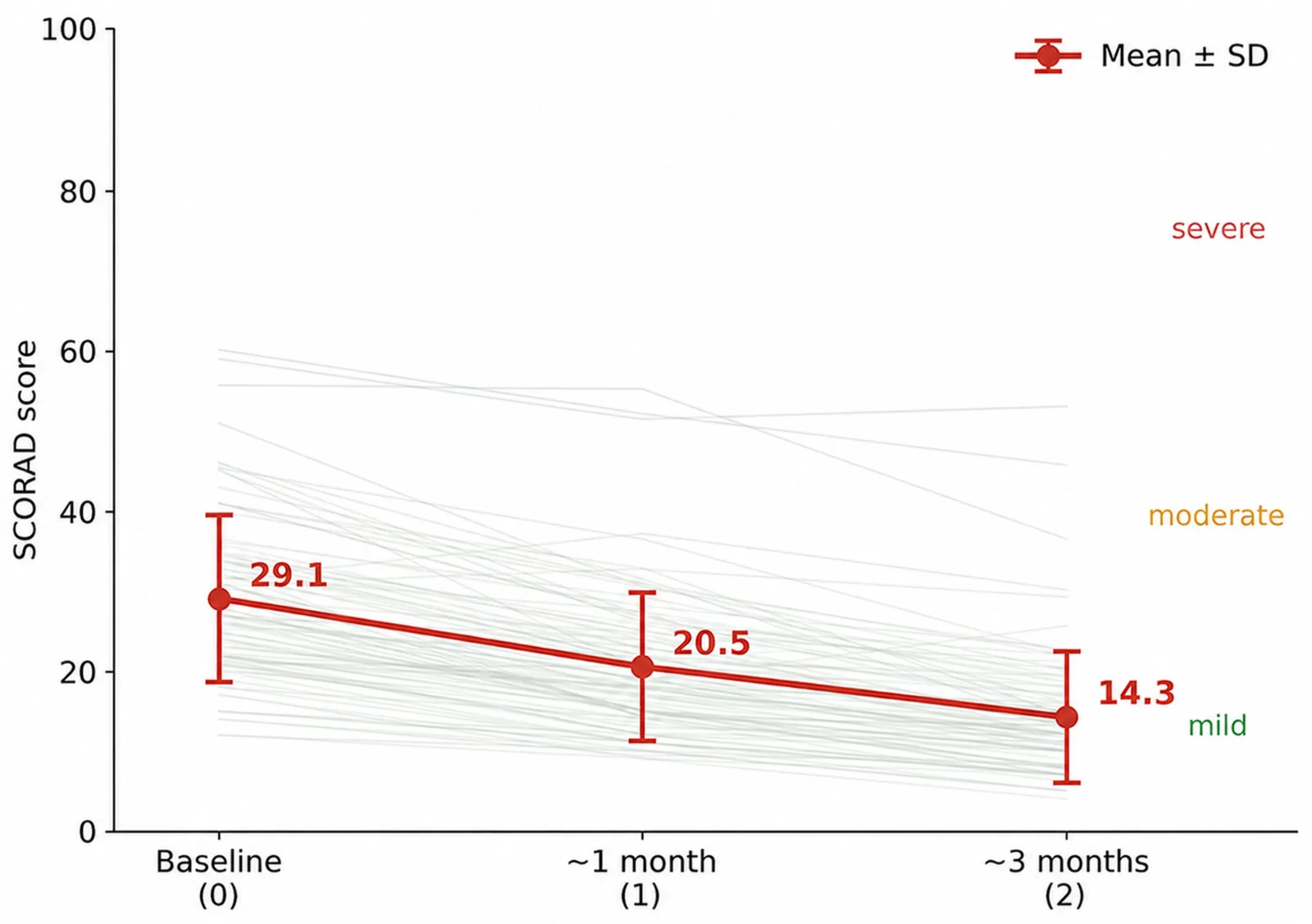
Longitudinal evolution of SCORAD during individualized nutritional and microbiome-oriented management. Thin grey lines represent individual participants, and the red line indicates the cohort mean ± SD. Background shading denotes mild (<25), moderate (25–50), and severe (>50) disease categories.

Clinical improvement was observed throughout follow-up, with progressive reductions in disease severity across the three assessments. Mean SCORAD declined from 29.1 (95% CI 26.8–31.3) at baseline to 20.6 (95% CI 18.6–22.6) after approximately one month and 14.3 (95% CI 12.5–16.0) after approximately three months. In the primary linear mixed-effects model, SCORAD decreased by an estimated 7.8 points (95% CI 6.5–9.1) at one month and 13.6 points (95% CI 12.3–14.9) at three months compared with baseline (both *p* < 0.001). These findings were confirmed by the Friedman test (χ^2^ = 158.2, *p* < 0.001), with all pairwise Wilcoxon signed-rank comparisons remaining significant after Bonferroni correction (Table 4, Figure 1).

**Figure 2:**
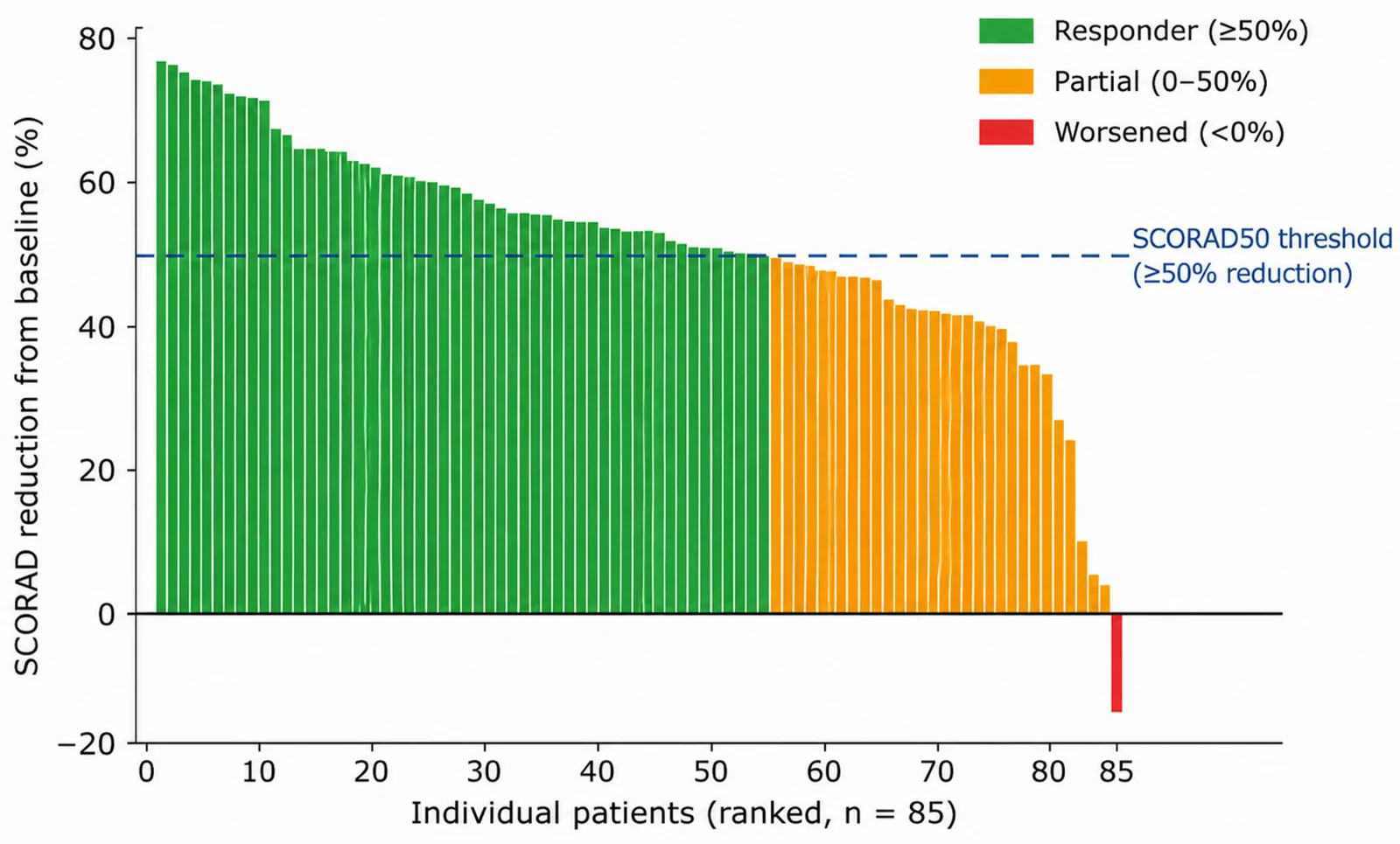
Waterfall plot showing the percentage change in SCORAD from baseline to three months. Green bars indicate SCORAD50 responders (≥50% reduction), orange bars partial responders, and the red bar the single participant with worsening disease.

The mean absolute improvement from baseline to three months was 14.8 SCORAD points (95% CI 13.3–16.3), corresponding to a mean relative reduction of 51.6% (95% CI 48.1–55.1). A SCORAD50 response (≥50% reduction) was achieved by 54 of 85 children (63.5%; 95% CI 52.9–73.0), whereas only one child (1.2%) experienced a net increase in SCORAD during follow-up (Figure 2). Sensitivity analyses yielded virtually identical results, confirming the robustness of the observed clinical improvement. In the absence of a concurrent control group, these longitudinal changes describe the clinical evolution observed under individualized management and cannot be attributed solely to the intervention.

### 3.4. Gut Dysbiosis, Food Allergy Burden, and Clinical Response

#### 3.4.1. Food Allergy Burden

**Figure 3:**
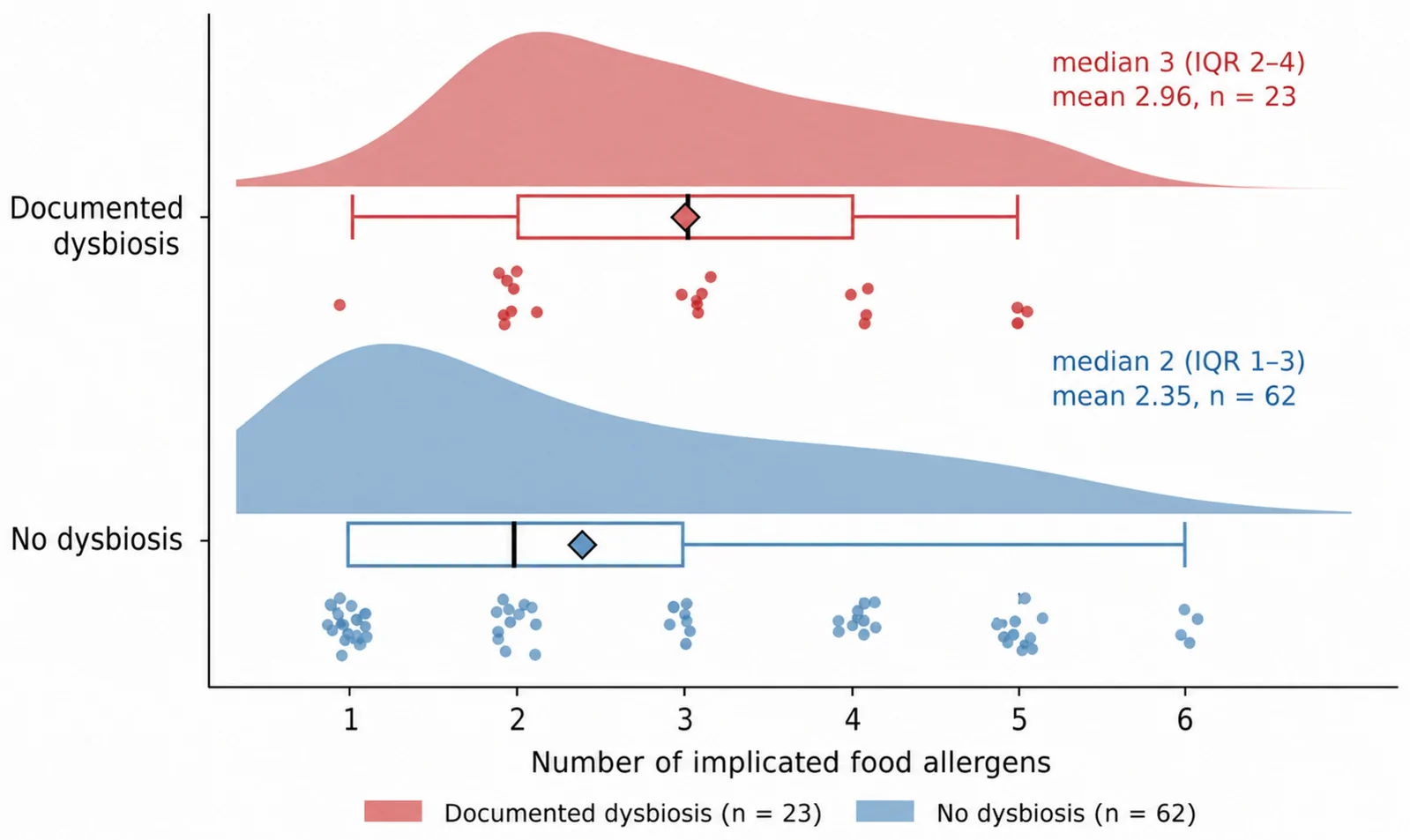
Raincloud plot showing the distribution of the number of implicated food allergens according to gut dysbiosis status. Half-violins represent the density distribution, box plots indicate the median and interquartile range (diamond = mean), and jittered points represent individual participants.

Documented gut dysbiosis was identified in 23 children (27.1%). Children with documented gut dysbiosis had a greater food allergy burden than those without documented gut dysbiosis. They were sensitized to a higher number of food allergens (median 3 [IQR 2–4] vs. 2 [1,2,3]; Mann–Whitney U, *p* = 0.028; rank-biserial correlation = 0.30) and were more frequently polyallergic (95.7% vs. 59.7%; χ^2^ = 8.60, *p* = 0.003; Fisher’s exact *p* = 0.001; Cramér’s *V* = 0.32) (Figure 3). In contrast, the distribution of allergy mechanisms (IgE-mediated, non-IgE-mediated, or mixed) did not differ according to dysbiosis status (χ^2^ = 1.95, *p* = 0.38).

Concomitant therapy was generally comparable between groups. Topical corticosteroids (21.7% vs. 27.4%; *p* = 0.78), topical calcineurin inhibitors (30.4% vs. 29.0%; *p* = 1.00), and probiotics (approximately 95% in both groups) were used at similar frequencies. Children with documented gut dysbiosis received omega-3 polyunsaturated fatty acids more frequently (47.8% vs. 21.0%; *p* = 0.028) and vitamin D_3_ less frequently (56.5% vs. 87.1%; *p* = 0.005).

#### 3.4.2. Clinical Response

Children with documented gut dysbiosis had higher baseline SCORAD scores than those without dysbiosis but achieved comparable SCORAD values after approximately three months of follow-up (mean 13.9 vs. 14.4, *p* = 0.62). They also showed greater absolute improvement in disease severity (mean ΔSCORAD 18.1 vs. 13.6 points; Mann–Whitney U, *p* = 0.006, q = 0.029), whereas the difference in relative percentage improvement did not reach statistical significance (55.7% vs. 50.1%; *p* = 0.077) (Table 5).

**Figure 4:**
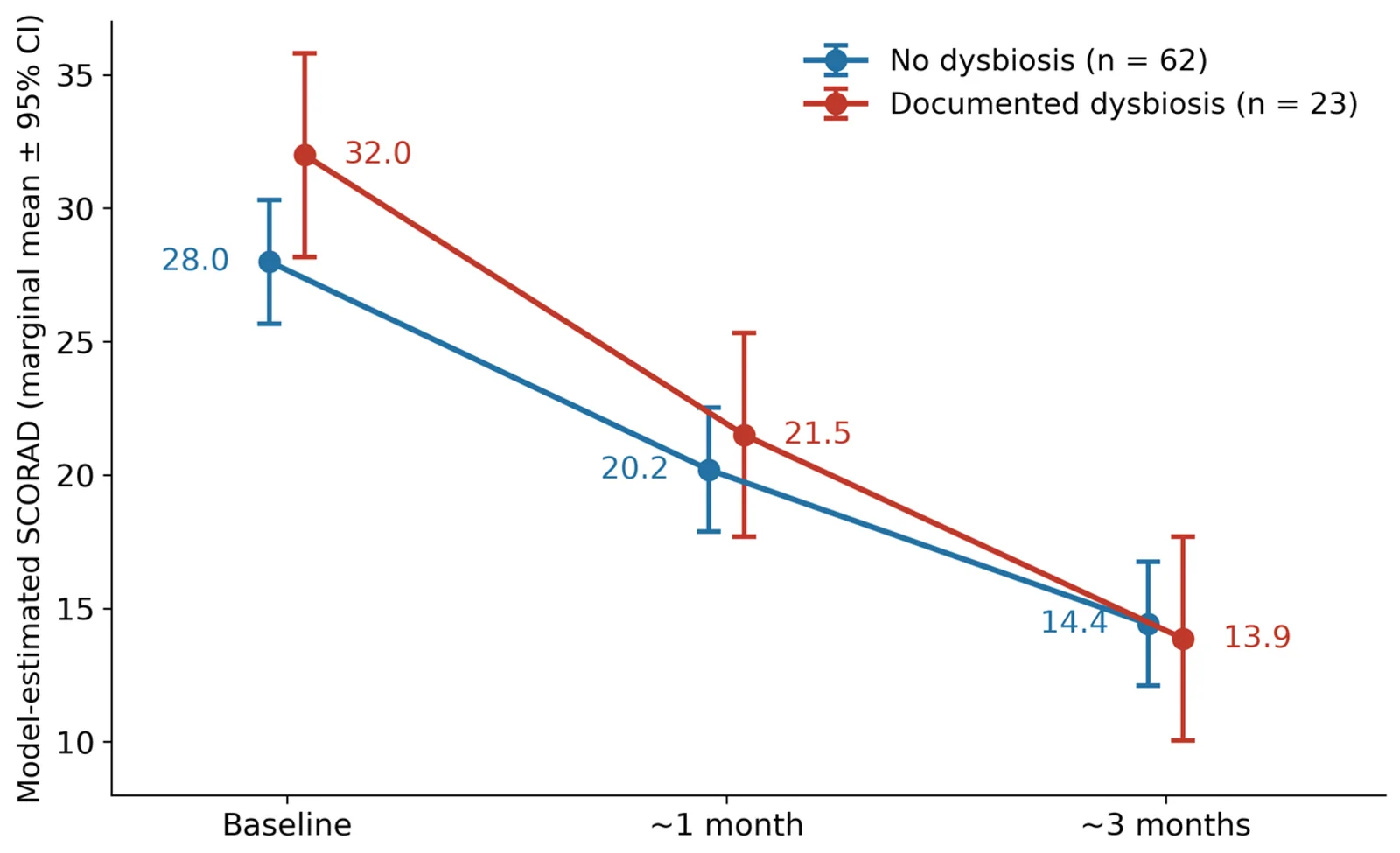
Model-estimated SCORAD trajectories according to documented gut dysbiosis status. Estimated marginal means (±95% CI) were derived from the linear mixed-effects model with a random intercept for each participant.

The linear mixed-effects model confirmed a significantly steeper decline in SCORAD among children with documented gut dysbiosis, with additional reductions of 2.7 points at approximately one month (95% CI 0.2–5.2, *p* = 0.036) and 4.6 points at approximately three months (95% CI 2.0–7.1, *p* < 0.001) compared with children without dysbiosis (Figure 4, Table 6). Bootstrap analyses confirmed the robustness of these findings.

The magnitude of absolute clinical improvement also differed according to food allergy burden. Children with a greater number of implicated food allergens achieved larger reductions in SCORAD (Kruskal–Wallis *p* = 0.013, q = 0.042), whereas relative percentage improvement and improvement according to allergy mechanism did not differ significantly.

### 3.5. Multivariable Predictors of Clinical Improvement

To identify independent predictors of clinical improvement, three pre-specified multivariable models were fitted, evaluating final SCORAD (primary analysis), absolute improvement (ΔSCORAD; complementary analysis), and SCORAD50 response (Table 7). All models were adjusted for baseline SCORAD, documented gut dysbiosis, number of implicated food allergens, age, sex, and concomitant topical corticosteroid and calcineurin inhibitor use. No evidence of relevant multicollinearity was observed (all VIF < 1.3).

In the primary analysis of covariance for final SCORAD (Model 2; adjusted R^2^ = 0.58), higher baseline SCORAD independently predicted higher final disease severity (β = 0.56, 95% CI 0.41–0.71; *p* < 0.001). Documented gut dysbiosis was associated with a lower final SCORAD (β = −2.69, 95% CI −5.92 to 0.54), although the association did not reach statistical significance (*p* = 0.10). Neither topical corticosteroid nor topical calcineurin inhibitor use was independently associated with final SCORAD.

**Figure 5:**
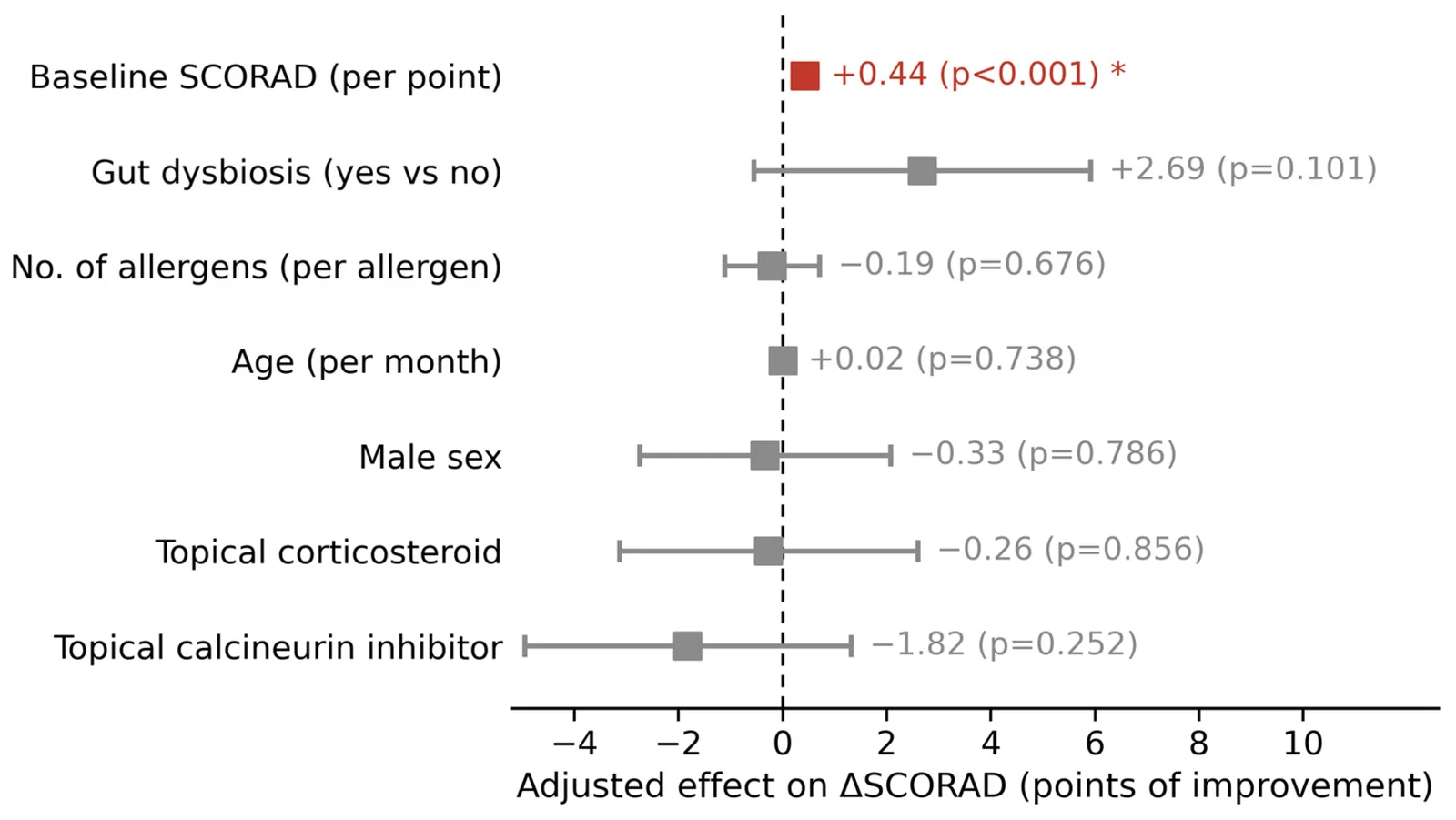
Forest plot of the complementary multivariable model for absolute clinical improvement (ΔSCORAD; Model 3, n = 85); *, statistically significant.

The complementary model evaluating absolute clinical improvement (Model 3; adjusted R^2^ = 0.39) yielded consistent findings. Baseline SCORAD remained the strongest independent predictor of greater improvement (β = 0.44, standardized β = 0.68, *p* < 0.001), while documented gut dysbiosis was associated with an additional 2.69-point reduction in SCORAD (95% CI −0.54 to 5.92), although this association was not statistically significant after multivariable adjustment (*p* = 0.10). Bootstrap analyses yielded consistent estimates (Figure 5). Accordingly, the greater improvement observed among children with documented gut dysbiosis was largely explained by their higher baseline disease severity, and dysbiosis itself should not be interpreted as a favorable prognostic factor or as evidence of a direct therapeutic effect of microbiome-directed interventions.

In the logistic regression model for SCORAD50 response (Model 4), none of the evaluated baseline variables was independently associated with responder status. Documented gut dysbiosis showed the largest estimated effect (OR 2.26, 95% CI 0.62–8.29, *p* = 0.22), whereas baseline SCORAD, number of implicated food allergens, and age were not significantly associated with the probability of achieving a SCORAD50 response (Nagelkerke R^2^ = 0.07; AUC = 0.63).

### 3.6. Exploratory Analysis of Systemic Adjuvant Therapies

The systemic component of individualized management comprised probiotic/synbiotic preparations (95.3%), vitamin D_3_ (78.8%), oral antihistamines (47.1%), omega-3 polyunsaturated fatty acids (28.2%), prebiotic inulin (23.5%), and, less frequently, iron (10.6%), zinc, or antiparasitic agents (3.5% each). Children received a median of three systemic agents. The number of systemic agents was not associated with the magnitude of clinical improvement (Spearman’s ρ = −0.06, *p* = 0.57).

In univariate analyses, none of the evaluated systemic adjuvant therapies was associated with greater absolute clinical improvement. Omega-3 polyunsaturated fatty acids showed a non-significant trend toward greater improvement (ΔSCORAD 16.5 vs. 14.1; *p* = 0.084), whereas prebiotic inulin was associated with smaller absolute improvement (ΔSCORAD 12.2 vs. 15.6; *p* = 0.034). Oral antihistamine use was associated with a lower likelihood of achieving a SCORAD50 response (OR 0.28; *p* = 0.012) (Table 8).

In the multivariable model adjusted for baseline SCORAD, documented gut dysbiosis, and the four most frequently used systemic adjuvant therapies (adjusted R^2^ = 0.44), baseline SCORAD (β = 0.40, *p* < 0.001) and documented gut dysbiosis (β = 3.01, 95% CI 0.25–5.77; *p* = 0.033) remained independently associated with greater absolute clinical improvement. None of the evaluated systemic adjuvant therapies independently predicted improvement, with the exception of prebiotic inulin, which retained a negative association (β = −2.95, 95% CI −5.62 to −0.27; *p* = 0.031). Probiotic supplementation was not evaluated because it was prescribed to almost all participants (95.3%).

### 3.7. Clinical Improvement According to Baseline Disease Severity

**Figure 6:**
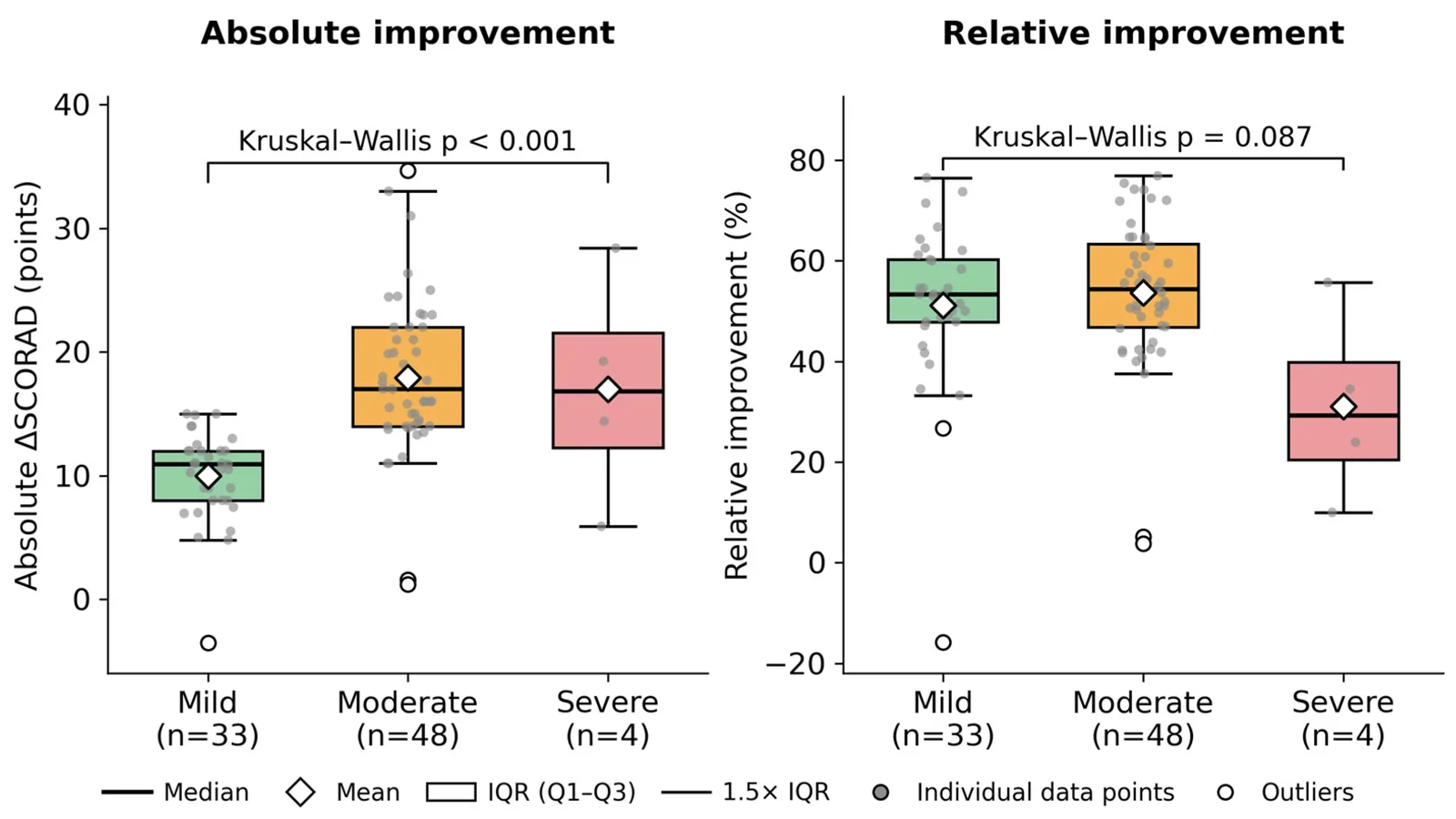
Absolute and relative improvement in SCORAD according to baseline disease severity. Boxplots show the median and interquartile range, diamonds indicate group means, and dots represent individual participants.

Clinical improvement was observed across all baseline disease severity categories. Children with mild disease improved from a mean SCORAD of 19.6 to 9.6 (mean ΔSCORAD 10.0; 51.2% relative improvement; 63.6% SCORAD50 responders), whereas children with moderate disease improved from 33.3 to 15.3 (mean ΔSCORAD 17.9; 53.6% relative improvement; 66.7% responders). Only four children had severe disease at baseline; therefore, outcomes for this subgroup are presented descriptively (mean ΔSCORAD 17.0; 31.0% relative improvement). Absolute improvement differed significantly across baseline severity groups (Kruskal–Wallis *p* < 0.001), whereas relative improvement did not (*p* = 0.087) (Figure 6).

Disease severity categories shifted markedly during follow-up. Among the 48 children with moderate baseline disease, 46 (95.8%) were classified as having mild disease after three months. Overall, the proportion of children with mild disease increased from 38.8% at baseline to 92.9% at follow-up (79 mild, 5 moderate, and 1 severe), representing a significant change in disease severity distribution (Bowker test, *p* < 0.001).

## 4. Discussion

In this prospective, two-center, real-world observational cohort of 85 young children with atopic dermatitis (AD) and confirmed food allergy, individualized nutritional and microbiome-oriented management was associated with substantial clinical improvement over approximately three months, with nearly two-thirds of children achieving a SCORAD50 response. Improvement was observed across all baseline severity categories, although children with more severe disease experienced greater absolute benefit. Beyond the overall therapeutic response, food allergy burden emerged as the principal determinant of baseline disease severity, while documented gut dysbiosis identified a subgroup with more severe allergic disease that experienced greater absolute clinical improvement during follow-up. Collectively, these findings support the concept that interactions between food allergy, gut dysbiosis, and skin inflammation contribute to the clinical heterogeneity of pediatric AD and reinforce the rationale for integrated nutritional and microbiome-oriented management within the gut–skin axis [38,39,40,41]. Given the observational, uncontrolled design, the associations described below are descriptive and should not be interpreted as causal treatment effects.

The allergological profile of the cohort was consistent with the epidemiology of food allergy-associated AD in early childhood, with cow’s milk protein, egg, and wheat representing the predominant allergens and more than two-thirds of children presenting with polyallergy. This pattern reflects the predominance of staple food allergens during infancy and highlights the nutritional challenges associated with eliminating multiple staple foods. These findings emphasize the importance of individualized nutritional assessment and appropriate dietary substitution to maintain adequate nutrient intake and support normal growth [42,43,44]. When cow’s milk must be eliminated, guideline-endorsed substitutes in formula-fed infants are extensively hydrolyzed and amino acid-based formulas, whereas unmodified milk from other ruminants such as goat or sheep is not recommended because of extensive cross-reactivity with cow’s milk proteins [10,37]. Beyond their nutritional composition, milks from different mammalian species are increasingly being investigated as sources of bioactive molecules with potential immunomodulatory properties. Recent studies have identified extracellular-vesicle-associated microRNAs in animal milks, including conserved miRNAs capable of remaining detectable following simulated gastrointestinal digestion and predicted to regulate immune-related pathways [45,46]. These observations raise interest in the potential biological relevance of species-specific milk-derived components; however, their clinical significance in children with food allergy or AD remains to be established. Importantly, such emerging mechanistic evidence should not be interpreted as supporting the unrestricted use of alternative mammalian milks in cow’s-milk protein allergy, because immunological cross-reactivity may occur. Current nutritional management therefore continues to favor extensively hydrolyzed or, when clinically indicated, amino acid-based formulas [10,37].

The predominance of non-IgE-mediated food allergy further illustrates the heterogeneous immunopathology of pediatric AD and reinforces the need for an individualized diagnostic approach integrating clinical history with appropriate allergological investigations. Consistent with current international guidelines, dietary interventions should be based on confirmed food allergy to avoid unnecessary elimination diets and their associated nutritional risks [47].

Food allergy burden emerged as the principal determinant of baseline disease severity, with each additional implicated food allergen associated with a significant increase in SCORAD. This finding is consistent with previous reports showing that children with multiple food allergies are more likely to present with severe eczema and greater immune dysregulation. Biologically, progressive epidermal barrier dysfunction facilitates sensitization to multiple dietary antigens, while persistent type 2 inflammation further compromises barrier integrity, creating a self-perpetuating cycle that sustains allergic inflammation and disease severity [48]. Clinically, these findings emphasize the importance of comprehensive nutritional assessment, as elimination of multiple staple foods, particularly milk, egg, and wheat, requires appropriate dietary substitution to maintain adequate nutrient intake and support normal growth [49].

Documented gut dysbiosis was associated with a greater food allergy burden and a markedly higher prevalence of polyallergy, although the distribution of IgE-mediated, non-IgE-mediated, and mixed allergy phenotypes was similar between groups. These findings are consistent with evidence that gut microbial dysbiosis contributes to impaired oral tolerance and allergic sensitization through disruption of epithelial barrier integrity, reduced production of short-chain fatty acids, and impaired regulatory T-cell function [50,51]. Importantly, emerging longitudinal and mechanistic evidence suggests that alterations in the gut microbiota may precede the clinical onset of food allergy and contribute to impaired oral tolerance, rather than merely accompany established allergic disease [40,52].

Rather than being associated with a specific immunological phenotype, documented gut dysbiosis appeared to reflect the overall burden of allergic disease, supporting the concept that microbial imbalance may represent a common pathway underlying multiple forms of food allergy. Although children with documented gut dysbiosis presented with more severe disease at baseline, they experienced greater absolute clinical improvement during follow-up. This association remained apparent in longitudinal and sensitivity analyses but was attenuated after adjustment for baseline disease severity and concomitant topical therapy and was no longer statistically significant in the fully adjusted model (*p* = 0.10). The greater observed improvement in children with documented dysbiosis was therefore largely explained by their higher baseline disease severity rather than by dysbiosis itself. Accordingly, dysbiosis should not be interpreted as a favorable prognostic factor or as evidence of a direct therapeutic effect of microbiome-directed interventions. These findings should therefore be regarded as hypothesis-generating rather than evidence of causality. Nevertheless, the consistency of the findings suggests that gut dysbiosis may represent a clinically relevant stratification factor for integrated nutritional and microbiome-oriented management. Confirmation in larger prospective studies incorporating sequencing-based microbiome profiling and functional microbial analyses is warranted. In contrast, the Flora Index was not associated with disease severity within the dysbiosis subgroup, indicating that the clinical identification of gut dysbiosis may currently have greater prognostic value than quantification using this culture-based index [39,41].

This study evaluated the real-world effectiveness of integrated nutritional and microbiome-oriented management rather than the efficacy of individual dietary interventions. Management combined targeted elimination diets, microbiome-oriented interventions, micronutrient supplementation, conventional topical therapy, and personalized nutritional counselling according to each child’s clinical presentation, allergological profile, gastrointestinal manifestations, and stool microbiological findings. Consequently, the observed clinical improvement should be interpreted as the effect of this integrated approach rather than any single therapeutic component. This distinction is clinically relevant because children with food allergy-associated AD are typically managed using multimodal strategies that combine nutritional, allergological, microbiological, and dermatological interventions tailored to individual clinical needs [37,53].

This integrated, patient-centred approach is consistent with the growing shift toward precision medicine and personalized nutritional management in pediatric allergic diseases, where therapeutic decisions increasingly consider individual clinical characteristics, underlying biological mechanisms, and disease heterogeneity [54]. A similar individualized paradigm is increasingly adopted across pediatric practice, including the use of targeted genetic testing to guide the diagnosis and management of nutrition-related disorders such as familial hypercholesterolemia [55].

The available evidence provides biological plausibility for the integrated nutritional strategy evaluated in this study. Vitamin D_3_ has well-established immunomodulatory effects and supports epidermal barrier function, while recent randomized trials and systematic reviews have reported modest improvements in pediatric AD [56,57]. Likewise, probiotic and synbiotic supplementation has demonstrated modest but consistent benefits, particularly with multi-strain preparations containing *Lactobacillus* and *Bifidobacterium* species administered during early childhood. The clinical improvement observed in the present cohort is broadly consistent with previous evidence suggesting potential benefits of microbiome- and nutrition-oriented interventions in pediatric AD. Meta-analyses of randomized controlled trials have reported reductions in SCORAD following multi-strain probiotic supplementation in children with AD [21,22], while recent meta-analytic evidence also suggests an overall beneficial effect of vitamin D supplementation on AD severity [24]. However, these findings cannot be directly compared with the magnitude of improvement observed in our cohort because the present intervention was individualized and multimodal, probiotics were prescribed to nearly all participants, and the study lacked a concurrent control group. Accordingly, our findings do not establish the independent efficacy of probiotics, vitamin D, or any other individual component of the management strategy. Similarly, omega-3 polyunsaturated fatty acids possess anti-inflammatory and immunomodulatory properties, although evidence supporting their independent clinical benefit remains heterogeneous. Because these interventions were implemented as components of an integrated nutritional strategy, the present study was not designed to determine their individual therapeutic effects but rather provides real-world evidence supporting their combined implementation in routine pediatric practice [58,59,60]. The potential contribution of individual micronutrients also deserves consideration. Zinc contributes to epidermal barrier integrity, keratinocyte differentiation, and innate and adaptive immune function, while lower serum zinc concentrations have been reported in infants with AD and associated with disease phenotype and severity [57]. Iron status is similarly relevant in children with allergic disease, particularly when multiple nutritionally important foods are eliminated, as inadequate dietary substitution may increase the risk of iron and zinc deficiencies [11,12]. Vitamin D contributes to cutaneous antimicrobial defense, epidermal barrier function, and immune regulation, including regulatory T-cell responses, and recent meta-analytic evidence suggests that supplementation may improve AD severity [24,56]. In the present cohort, documented iron (10.6%) and zinc (3.5%) deficiencies were addressed through individualized supplementation; however, micronutrient status was not systematically reassessed during follow-up. Consequently, biochemical correction of these deficiencies could not be confirmed, and no conclusion can be drawn regarding the independent contribution of micronutrient supplementation to the observed clinical improvement.

The multivariable analyses further refined the interpretation of these findings. Baseline SCORAD consistently emerged as the strongest independent predictor of subsequent absolute clinical improvement, indicating that children with more severe disease experienced greater absolute benefit during follow-up. Although this observation partly reflects the greater potential for improvement among patients with higher baseline disease activity, it is also consistent with the greater absolute improvements typically observed among patients with moderate-to-severe AD receiving effective therapy [2,61]. Gut dysbiosis likewise remained positively associated with greater clinical improvement across multiple analytical approaches, although this association was attenuated after adjustment for baseline disease severity and concomitant treatment. These findings should therefore be considered hypothesis-generating rather than evidence of an independent causal relationship [41,62].

Several clinically relevant variables were not independently associated with baseline disease severity or longitudinal clinical response. Neither cesarean delivery, prematurity, breastfeeding history, nor allergy mechanism remained significant after adjustment for potential confounders. Likewise, although children with a greater food allergy burden experienced larger absolute improvements, relative improvement was comparable across allergy burden categories and immunological phenotypes. These findings suggest that, once food allergy-associated AD is clinically established, baseline disease severity may be a stronger determinant of short-term clinical response than early-life exposures or the underlying allergy mechanism. However, because perinatal factors have consistently been associated with the development of allergic diseases and the atopic march in population-based studies, the absence of significant associations in the present cohort should be interpreted cautiously and may reflect the modest sample size and limited statistical power [63].

Because the Flora Index summarizes conventional microbiological findings rather than sequencing-derived microbial diversity or taxonomic composition, it should not be interpreted as a comprehensive measure of gut microbiome complexity. Nevertheless, it illustrates the potential utility of standardized stool microbiology to support personalized nutritional decision-making in routine clinical practice, particularly where sequencing-based technologies are not readily available [64,65].

Together, these findings support the growing shift toward personalized nutritional management in pediatric allergy. Rather than applying uniform dietary recommendations, integrated strategies combining allergological assessment, personalized nutritional counselling, microbiome evaluation, and conventional dermatological treatment may better address the marked heterogeneity of food allergy-associated AD. Although this approach requires confirmation in randomized controlled trials, it aligns with the emerging paradigm of precision nutrition, which aims to tailor dietary interventions according to individual clinical and biological characteristics [18,66].

The very young age of the cohort further underscores the importance of early intervention, as infancy represents a critical window for immune maturation, gut microbiome development, and epithelial barrier establishment [43,44].

Several limitations should be considered when interpreting the present findings. The prospective observational cohort design without a control group precludes causal inference and does not allow the effects of the individualized nutritional intervention to be fully separated from spontaneous improvement or regression toward the mean. The multimodal management strategy reflects routine clinical practice but precludes attribution of benefit to individual therapeutic components. Food allergy was diagnosed using clinical history supported by skin prick testing, atopy patch testing, and allergen-specific IgE measurements rather than double-blind placebo-controlled oral food challenges; therefore, food allergy should be regarded as clinically probable rather than challenge-proven, and some degree of diagnostic misclassification cannot be excluded. Stool testing was also limited to children with gastrointestinal manifestations suggestive of dysbiosis rather than being performed systematically across the entire cohort, introducing potential selection bias; consequently, all dysbiosis-related findings should be regarded as exploratory. In particular, weight, height, body mass index (BMI), and growth-curve percentiles were not systematically recorded as study outcomes, and follow-up biochemical testing (e.g., serum iron, ferritin, zinc, and calcium) was not performed; consequently, the nutritional safety of the elimination diets, most importantly the withdrawal of cow’s milk in 92.9% and of wheat in 37.6% of participants, could not be objectively verified, and subclinical growth impairment or micronutrient deficiencies cannot be excluded. Because probiotics were prescribed to 95.3% of participants, the independent effect of microbiome-directed supplementation could not be evaluated, and repeated SCORAD assessments were not subjected to formal inter-rater reliability testing. Moreover, the culture-based Flora Index cannot capture overall microbial community structure; sequencing-based approaches (16S rRNA gene sequencing or shotgun metagenomics) with alpha- and beta-diversity analyses would be required for comprehensive taxonomic characterization. Finally, the relatively modest sample size, particularly for severe AD, and the approximately three-month follow-up limit assessment of long-term effectiveness and potential effects on the atopic march.

Despite these limitations, the study has several important strengths. It represents one of the few prospective real-world observational cohort studies evaluating an integrated nutritional and microbiome-oriented management strategy in children with confirmed food allergy-associated AD. Standardized clinical assessment at three predefined time points, together with mixed-effects modelling, multivariable analyses, responder analyses, bootstrap resampling, false-discovery-rate correction, and sensitivity analyses, consistently supported the robustness of the findings. The study also provides clinically relevant effect-size estimates that may inform the design of future randomized controlled trials.

From a clinical perspective, these findings support the feasibility of integrating personalized nutritional counselling, targeted elimination diets, and microbiome-oriented interventions into routine pediatric care as adjuncts to conventional dermatological therapy. Rather than replacing standard treatment, this integrated approach may complement existing care by simultaneously addressing food allergy, nutritional status, gastrointestinal manifestations, and gut dysbiosis. Pending confirmation in controlled trials, several pragmatic dietary principles may be considered for children with food allergy-associated AD. Elimination should be restricted to foods with confirmed clinical relevance, avoiding empirical broad elimination diets. In formula-fed infants with cow’s milk protein allergy, extensively hydrolyzed or, when indicated, amino acid-based formulas should be used rather than unmodified mammalian milks or nutritionally incomplete plant-based drinks [10,37]. Adequate nutritional substitution should be ensured, with particular attention to energy, protein, calcium, iron, zinc, and vitamin D intake, ideally under the supervision of a pediatric dietitian [11,12]. Food tolerance should be periodically reassessed to allow timely and safe reintroduction of eliminated foods [10,47], while growth should be monitored throughout dietary therapy. Future randomized controlled trials should compare this integrated management strategy with standard dermatological care while incorporating sequencing-based microbiome profiling, metabolomic analyses, comprehensive nutritional assessment (including systematic anthropometric monitoring with growth-curve percentiles and longitudinal biochemical evaluation of micronutrient status), and longer follow-up. Stratification according to gut dysbiosis status, food allergy burden, and immunological phenotype may help identify children most likely to benefit from personalized nutritional interventions and further advance precision nutrition strategies for pediatric AD [52].

Ultimately, integrating allergological assessment, personalized nutritional management, and microbiome characterization may contribute to more individualized therapeutic strategies capable of improving disease control during the earliest stages of the atopic march.

## 5. Conclusions

In this real-world, two-center cohort of young children with atopic dermatitis and confirmed food allergy, an individualized nutritional and microbiome-oriented management strategy combining elimination of the dominant food allergens with targeted microbiome modulation, micronutrient support, and standard dermatological care was associated with substantial and progressive improvement in disease severity, with nearly two-thirds of children achieving a SCORAD50 response. A greater food allergy burden was independently associated with more severe baseline disease, while children with documented gut dysbiosis, despite presenting with more severe disease and greater allergic burden, experienced greater absolute clinical improvement during follow-up. Together, these findings further support the clinical relevance of the gut–skin–food axis and highlight the potential value of individualized nutritional management as an adjunct to conventional therapy in children with food allergy-associated atopic dermatitis.

It must be emphasized that the observational, uncontrolled design of this study precludes any inference of causal treatment efficacy, and this integrated strategy cannot be considered clinically validated until its benefits have been confirmed in randomized controlled trials. Nevertheless, the present study provides clinically relevant real-world evidence and effect-size estimates that may inform the design and evaluation of future controlled studies of personalized nutritional strategies for pediatric atopic dermatitis.

## Figures and Tables

**Table 1:** Baseline demographic, clinical, microbiological, and perinatal characteristics.

Characteristic	Value
Age, months—mean ± SD	15.9 ± 12.4
Age, months—median (IQR); range	11.0 (6.0–24.0); 3–48
Age < 12 months—n (%)	43 (50.6)
Male sex—n (%)	45 (52.9)
Cesarean section—n (%)	63 (74.1)
Preterm birth—n (%)	14 (16.5)
In vitro fertilization—n (%)	4 (4.7)
Any breastfeeding—n (%)	49 (57.6)
Documented gut dysbiosis—n (%)	23 (27.1)
Flora Index (dysbiosis subgroup)—mean ± SD	5.4 ± 1.4
Number of food allergens—mean ± SD	2.5 ± 1.4
Polyallergic (≥2 allergens)—n (%)	59 (69.4)
Baseline SCORAD—mean ± SD	29.1 ± 10.4
Baseline severity: mild/moderate/severe—n (%)	33 (38.8)/48 (56.5)/4 (4.7)

IQR, interquartile range; SCORAD, Scoring Atopic Dermatitis; SD, standard deviation. Mild SCORAD < 25; moderate 25–50; severe > 50.

**Table 2:** Food allergy profile of the study cohort.

Characteristic	n	%
Food allergens		
Cow’s milk protein	79	92.9
Egg	49	57.6
Wheat/gluten	32	37.6
Tree nuts (including cashew and pistachio)	12	14.1
Potato	11	12.9
Banana	6	7.1
Rice	5	5.9
Oat	3	3.5
Soy	2	2.4
Fish	2	2.4
Other (mustard, coconut, sunflower, beef, avocado)	≤1 each	1.2
Food allergy burden		
Number of implicated food allergens, mean ± SD	2.5 ± 1.4	—
Monoallergic (1 food allergen)	26	30.6
Polyallergic (≥2 food allergens)	59	69.4
Allergy mechanism		
Non-IgE-mediated (delayed) only	51	60.0
IgE-mediated only	20	23.5
Mixed	14	16.5

SD, standard deviation. Percentages for individual food allergens are calculated using the entire study cohort (n = 85); children could have more than one implicated food allergen. Allergy type was classified according to the clinical and allergological assessment described in the Methods.

**Table 3:** Factors associated with baseline disease severity (baseline SCORAD).

Factor	Analysis	Effect Estimate	*p*-Value	q †
Number of implicated food allergens	Spearman correlation	ρ = 0.31	0.004	0.029
Age (months)		ρ = 0.02	0.840	0.840
Flora Index (dysbiosis subgroup, n = 23)		ρ = 0.10	0.640	0.710
Documented gut dysbiosis (yes vs. no)	Mann–Whitney U	Median SCORAD: 32.0 vs. 26.2	0.044	0.110
Cesarean section		Mean SCORAD: 30.1 vs. 26.0	0.120	0.200
Prematurity		Mean SCORAD: 33.0 vs. 28.3	0.190	0.270
Breastfeeding history		Mean SCORAD: 29.9 vs. 28.0	0.420	0.530
Number of implicated food allergens (adjusted) ‡	Multiple linear regression	β = 2.19 (95% CI 0.55–3.82)	0.009	—

† Benjamini–Hochberg false discovery rate (FDR)-adjusted *q*-values for univariate analyses. ‡ Multiple linear regression (*n* = 85; R^2^ = 0.17) adjusted for age, sex, documented gut dysbiosis, number of implicated food allergens, cesarean section, prematurity, and breastfeeding history. The number of implicated food allergens was the only variable independently associated with baseline SCORAD; all remaining covariates were non-significant (*p* > 0.18).

**Table 4:** Longitudinal changes in SCORAD during individualized nutritional and microbiome-oriented management.

Assessment/Comparison	Mean SCORAD ± SD	Mean Change (ΔSCORAD)	*p*-Value	Effect Size
Baseline	29.1 ± 10.4	—	—	—
1 month	20.6 ± 9.3	−8.5	<0.001	dz = 1.72
3 months	14.3 ± 8.2	−14.8		dz = 2.17
Overall longitudinal change	—	—		Kendall’s W = 0.93
Pairwise comparisons				
Baseline vs. 1 month	—	−8.5	<0.001 †	dz = 1.72
≈1 month vs. 3 months	—	−6.3		dz = 1.51
Baseline vs. 3 months	—	−14.8		dz = 2.17

Overall longitudinal differences were evaluated using the Friedman test (χ^2^ = 158.2, *p* < 0.001). Pairwise comparisons were performed using the Wilcoxon signed-rank test with Bonferroni correction. ΔSCORAD indicates the mean within-patient change from the preceding assessment (or from baseline, where indicated). † Bonferroni-adjusted *p*-values.

**Table 5:** Associations of documented gut dysbiosis with food allergy burden, clinical outcomes, and concomitant therapy.

Characteristic	Documented Gut Dysbiosis (n = 23)	No Gut Dysbiosis (n = 62)	*p*-Value (Effect Size)
Food allergy burden			
Number of implicated food allergens, median (IQR)	3 (2–4)	2 (1–3)	0.028 (rank-biserial = 0.30)
Polyallergy (≥2 food allergens), n (%)	22 (95.7)	37 (59.7)	0.003 (Cramér’s *V* = 0.32)
Allergy mechanism (IgE/delayed/mixed), %	30/48/22	21/65/15	0.380 (Cramér’s *V* = 0.15)
Clinical outcomes			
Baseline SCORAD, median	32.0	26.2	0.044
Final SCORAD, mean	13.9	14.4	0.620
ΔSCORAD, mean	18.1	13.6	0.006 (rank-biserial = 0.39)
Relative improvement (%), mean	55.7	50.1	0.077
SCORAD50 responders, n (%)	17 (73.9)	37 (59.7)	0.210
Concomitant therapy			
Topical corticosteroids, n (%)	5 (21.7)	17 (27.4)	0.780
Topical calcineurin inhibitors, n (%)	7 (30.4)	18 (29.0)	1.000
Omega-3 PUFA, n (%)	11 (47.8)	13 (21.0)	0.028
Vitamin D3, n (%)	13 (56.5)	54 (87.1)	0.005

IQR, interquartile range; PUFA, polyunsaturated fatty acids; SCORAD, Scoring Atopic Dermatitis. ΔSCORAD was calculated as the baseline minus the 3-month SCORAD score; positive values indicate clinical improvement. Probiotic supplementation is not shown because it was prescribed to approximately 95% of children in both groups.

**Table 6:** Linear mixed-effects model of SCORAD over time (random intercept per patient; 255 observations, 85 patients).

Fixed Effect	β (95% CI)	*p*-Value
Intercept (baseline, no dysbiosis)	27.99 (25.66, 30.31)	<0.001
Time: 1 month	−7.79 (−9.10, −6.48)	
Time: 3 months	−13.57 (−14.88, −12.26)	
Gut dysbiosis (at baseline)	4.00 (−0.47, 8.46)	0.079
Dysbiosis × time (1 month)	−2.69 (−5.21, −0.18)	0.036
Dysbiosis × time (3 months)	−4.55 (−7.06, −2.03)	<0.001

Negative coefficients indicate lower SCORAD values over time. Negative dysbiosis × time interaction terms indicate greater improvement among children with documented gut dysbiosis. Between-patient variance (random intercept) = 73.3.

**Table 7:** Multivariable models of clinical outcome (n = 85), each adjusted for baseline SCORAD, gut dysbiosis, number of allergens, age, sex, and topical corticosteroid and calcineurin-inhibitor use.

Predictor	Final SCORAD (ANCOVA)		Absolute Improvement ΔSCORAD		SCORAD50 Response	
	β (95% CI)	*p*-Value	β (95% CI)	*p*-Value	OR (95% CI)	*p*-Value
Baseline SCORAD	0.56 (0.41, 0.71)	<0.001	0.44 (0.29, 0.59)	<0.001	0.98 (0.94, 1.03)	0.39
Documented gut dysbiosis	−2.69 (−5.92, 0.54)	0.10	2.69 (−0.54, 5.92)	0.10	2.26 (0.62, 8.29)	0.22
Number of implicated food allergens	0.19 (−0.72, 1.11)	0.68	−0.19 (−1.11, 0.72)	0.68	0.83 (0.59, 1.17)	0.28
Age (months)	−0.02 (−0.14, 0.10)	0.74	0.02 (−0.10, 0.14)	0.74	1.00 (0.96, 1.05)	0.91
Male sex	0.33 (−2.08, 2.74)	0.79	−0.33 (−2.74, 2.08)	0.79	—	—
Topical corticosteroid	0.26 (−2.61, 3.13)	0.86	−0.26 (−3.13, 2.61)	0.86	—	—
Topical calcineurin inhibitor	1.82 (−1.32, 4.95)	0.25	−1.82 (−4.95, 1.32)	0.25	—	—
Model fit	Adjusted R^2^ = 0.58		Adjusted R^2^ = 0.39		Nagelkerke R^2^ = 0.07; AUC = 0.63	

ANCOVA, analysis of covariance; ΔSCORAD, baseline minus 3-month SCORAD (positive values indicate greater clinical improvement); SCORAD50, ≥50% reduction in SCORAD from baseline; OR, odds ratio; AUC, area under the receiver operating characteristic curve.

**Table 8:** Associations between systemic adjuvant therapies and absolute clinical improvement (ΔSCORAD).

Systemic Adjuvant	n (%)	Mean ΔSCORAD (Yes vs. No)	Univariate *p*-Value	Adjusted β (95% CI)	Adjusted *p*-Value
Probiotic/synbiotic	81 (95.3)	14.9 vs. 13.1	0.56	Not evaluated †	—
Vitamin D3	67 (78.8)	14.5 vs. 15.8	0.41	−1.14 (−4.09 to 1.81)	0.45
Oral antihistamine	40 (47.1)	14.7 vs. 14.9	0.90	−1.61 (−3.89 to 0.68)	0.17
Omega-3 PUFA	24 (28.2)	16.5 vs. 14.1	0.084	−1.17 (−3.82 to 1.48)	0.38
Prebiotic inulin	20 (23.5)	12.2 vs. 15.6	0.034	−2.95 (−5.62 to −0.27)	0.031
Iron	9 (10.6)	11.2 vs. 15.2	0.11	Not included	—
Zinc	3 (3.5)	—	—	Not included	—
Antiparasitic treatment	3 (3.5)	—	—	Not included	—

ΔSCORAD was calculated as the baseline minus the 3-month SCORAD score; positive values indicate greater clinical improvement. Adjusted β coefficients were derived from a multiple linear regression model including baseline SCORAD, documented gut dysbiosis, vitamin D_3_, oral antihistamines, omega-3 polyunsaturated fatty acids, and prebiotic inulin (adjusted R^2^ = 0.44). † Probiotic supplementation was not evaluated because it was prescribed to 95.3% of participants.

## Data Availability

The original contributions presented in this study are included in the article. Further inquiries can be directed to the corresponding authors.

## Abbreviations

The following abbreviations are used in this manuscript:

AD	Atopic dermatitis
APT	Atopy patch test
CI	Confidence interval
FDR	False discovery rate
FI	Flora Index
GI	Gastrointestinal
HPF	High putrefactive flora
IgE	Immunoglobulin E
IQR	Interquartile range
OR	Odds ratio
PUFA	Polyunsaturated fatty acids
RCT	Randomized controlled trial
SCFA	Short-chain fatty acids
SCORAD	Scoring Atopic Dermatitis
SCORAD50	≥50% reduction in Scoring Atopic Dermatitis score from baseline
SD	Standard deviation
Treg	Regulatory T cell
